# Neonatal resuscitation: evolving strategies

**DOI:** 10.1186/s40748-014-0003-0

**Published:** 2015-01-22

**Authors:** Payam Vali, Bobby Mathew, Satyan Lakshminrusimha

**Affiliations:** Department of Pediatrics (Neonatology), University at Buffalo, Buffalo, NY USA; Women and Children’s Hospital of Buffalo, 219 Bryant St, Buffalo, NY 14222 USA; Division of Neonatology, Department of Pediatrics, Women and Children’s Hospital of Buffalo, 219 Bryant St, Buffalo, NY 14222 USA

**Keywords:** Cord clamping, Meconium, Ventilation, Sustained inflation, Chest compressions, Epinephrine, Oxygen

## Abstract

Birth asphyxia accounts for about 23% of the approximately 4 million neonatal deaths each year worldwide (Black et al., Lancet, 2010, 375(9730):1969-87). The majority of newborn infants require little assistance to undergo physiologic transition at birth and adapt to extrauterine life. Approximately 10% of infants require some assistance to establish regular respirations at birth. Less than 1% need extensive resuscitative measures such as chest compressions and approximately 0.06% require epinephrine (Wyllie et al. Resuscitation, 2010, 81 Suppl 1:e260–e287). Transition at birth is mediated by significant changes in circulatory and respiratory physiology. Ongoing research in the field of neonatal resuscitation has expanded our understanding of neonatal physiology enabling the implementation of improved recommendations and guidelines on how to best approach newborns in need for intervention at birth. Many of these recommendations are extrapolated from animal models and clinical trials in adults. There are many outstanding controversial issues in neonatal resuscitation that need to be addressed. This article provides a comprehensive and critical literature review on the most relevant and current research pertaining to evolving new strategies in neonatal resuscitation. The key elements to a successful neonatal resuscitation include ventilation of the lungs while minimizing injury, the judicious use of oxygen to improve pulmonary blood flow, circulatory support with chest compressions, and vasopressors and volume that would hasten return of spontaneous circulation. Several exciting new avenues in neonatal resuscitation such as delayed cord clamping, sustained inflation breaths, and alternate vasopressor agents are briefly discussed. Finally, efforts to improve resuscitative efforts in developing countries through education of basic steps of neonatal resuscitation are likely to decrease birth asphyxia and neonatal mortality.

**Electronic supplementary material:**

The online version of this article (doi:10.1186/s40748-014-0003-0) contains supplementary material, which is available to authorized users.

## Introduction

**Figure 1:**
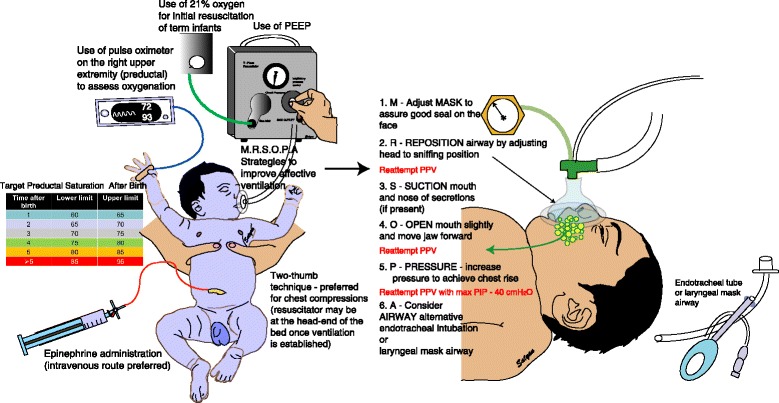
**Important changes to the neonatal resuscitation algorithm as per the 2011 guidelines.** (1) Recommendation of 21% oxygen for initial resuscitation of term infants and use of a blender to titrate inspired oxygen based on target preductal (right upper extremity pulse oximeter values – table); (2) Use of PEEP/CPAP preferably with a T-piece resuscitator; (3) Corrective steps to improve PPV by mask using the acronym “MR SOPA” – M – mask adjustment and R – reposition airway should be addressed first, then the next two steps (S – suction mouth and nose and O – open mouth). Then if there is not adequate chest movement, move to P – pressure increase and A – airway alternative with endotracheal intubation or laryngeal mask airway); (4) Use of two-thumb technique for chest compressions from the head-end of the bed to provide room for umbilical vein catheterization; and (5) early use of intravenous epinephrine. Copyright Satyan Lakshminrusimha.

Among more than 130 million births per year globally, approximately ten percent of newborns require some form of intervention immediately at birth. It is estimated that 25% of approximately 4 million neonatal deaths worldwide are secondary to birth asphyxia [1-4]. Unlike the emphasis on restitution of cardiac activity in adult resuscitation, the Working Group on Pediatric Resuscitation has recommended that resuscitation of newborns should focus on ventilation of the lungs [5]. By 1985, the American Academy of Pediatrics (AAP) and American Heart Association (AHA) expressed a joint commitment to develop a training program aimed at neonatal resuscitation. The first edition of the Neonatal Resuscitation Textbook was published in 1987. Since that time, the International Liaison Committee on Resuscitation (ILCOR) has continued to develop and publish a consensus on the science of resuscitation approximately every five years [6]. The initial recommendations were based predominantly on opinions from experts in the field. Through extensive research and review of literature in the past two decades, neonatal resuscitation guidelines are increasingly derived from experimental and experiential evidence from simulation manikins, animal models, randomized clinical trials [7-9], and systematic clinical observation [10]. However, certain aspects of the current guidelines remain controversial given the difficulties in conducting randomized clinical trials due to the infrequent and often unpredictable need for extensive resuscitation. Many research studies in neonatal resuscitation are conducted using postnatal, term animal models that fail to adequately depict the transitioning fetal circulation, an open ductus arteriosus, fluid-filled lungs and, in case of preterm birth, premature lungs. Deleterious effects associated with various steps of neonatal resuscitation such as hyperoxemia [10], barotrauma due to positive pressure ventilation (PPV), mechanical effects of chest compressions (CCs) and resuscitative medications [11] are being increasingly recognized. Hence, the guidelines for neonatal resuscitation focus on ventilation while establishing a functional residual capacity (FRC) without causing lung injury, optimizing oxygen delivery to the tissues without inducing toxicity, and hastening return of spontaneous circulation with effective CCs and drug delivery. The physiological basis of current and planned resuscitative measures is outlined below. A summary of important changes in the current version of neonatal resuscitation guidelines is shown in Figure 1.

## Review

### Fetal circulation, transition at birth, delayed (Physiological) Cord Clamping and Temperature Control

**Figure 2:**
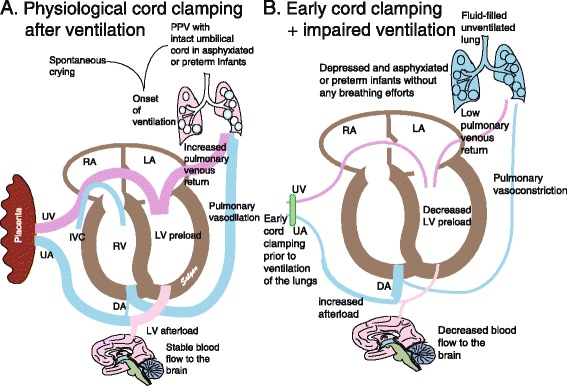
**Effects of cord clamping on hemodynamics.** An intact umbilical cord allows continuous umbilical venous flow to the ventricles. With the concomitant initiation of breathing through crying or PPV, PVR decreases allowing increased blood flow to the lungs (decreased right to left shunt through the DA) as well as increased venous return to the LV. The unclamped UA prevents a sudden increase in afterload. This results in improved cardiac output **(A)**. Conversely, immediate cord clamping restricts flow to the ventricles. With failure to establish ventilation, PVR remains high and compromises pulmonary blood flow (increased right to left DA shunt) and venous return to the left ventricle. Thus, decreased filling of the left ventricle (preload) and increased afterload (due to removal of low-resistance placenta) compromise cardiac output **(B)**. DA ductus arteriosus, PPV positive pressure ventilation, LA left atrium, RA right atrium, LV left ventricle, UA umbilical artery, UV umbilical vein. Copyright Satyan Lakshminrusimha.

Relatively oxygen-rich blood (PaO_2_ 20-40 mm Hg) from the umbilical vein entering the inferior vena cava (IVC) tends to stream and does not completely mix with the less oxygenated blood that enters the IVC from the lower half of the body (Figure 2A). This oxygen-rich blood is then preferentially diverted to the left ventricle through the foramen ovale to the left atrium. The fetal pulmonary venous return is low due to high pulmonary vascular resistance. Hence, the umbilical venous return is the chief component of left ventricular preload and determines oxygen delivery to the brain and heart (Figure 2A) [12]. In the human fetus, umbilical blood flow increases proportional to fetal weight gain and remains constant around 110-125 mL/min/kg. The fetal biventricular cardiac output is approximately 450 mL/min/kg and, thus, umbilical blood flow represents about 30% of the cardiac output [13].

The practice of early umbilical cord clamping started in the 20th century with an increasing number of hospital births and deliveries being conducted by obstetricians. In spite of little evidence or physiologic rationale to support immediate cord clamping, it remains the standard practice in the United States [14,15]. Numerous studies on delayed cord clamping have indicated benefits in improved hemodynamics, higher hematocrit levels, reduced need for blood transfusions and reduced incidence of intraventricular hemorrhage [16-20]. Delayed cord clamping and placental transfusion are associated with increased bilirubin levels without an increased need for phototherapy in preterm infants [20]. In term infants, there is evidence to suggest that delayed cord clamping is associated with increased need for phototherapy [21]. However, there is inconsistency with this association, as many large trials do not show a link between the need for phototherapy and delayed cord clamping [18,22]. Based on the current evidence, the benefits of delayed cord clamping appear to outweigh the risks associated with placental transfusion.

Term, non-asphyxiated, infants cry and initiate ventilation soon after birth prior to clamping the umbilical cord (Figure 2A). A recent study investigating the physiologic effects of ventilation on cord clamping in preterm lambs shows a significant improvement in cardiovascular stability if ventilation is commenced prior to cord clamping [23]. Umbilical cord clamping prior to ventilation of the lungs causes cessation of umbilical venous flow to the heart resulting in an abrupt drop in left ventricular filling volume, while occlusion of the umbilical artery increases left ventricular afterload (Figure 2B). In the absence of ventilation, pulmonary vascular resistance remains high resulting in decreased pulmonary blood flow and return of blood back to the left ventricle. Therefore, in the absence of established ventilation, cord clamping puts a significant strain on the newborn’s cardiovascular system to overcome increased afterload in the setting of decreased ventricle filling volume [14,23]. “Delaying” cord clamping until ventilation is established is “physiologic” cord clamping.

Due to growing evidence to support the beneficial effects of delayed cord clamping, ILCOR currently recommends that the cord should not be cut for at least one minute in infants not requiring resuscitation and the ACOG advocates delayed umbilical cord clamping in preterm neonates when feasible [24,25]. It is important to emphasize the importance of improved prognosis in newborns allowed to undergo a smooth transition at birth by delaying cord clamping until ventilation (spontaneous or PPV) of the lungs is established (Figure 2).

Newborns, particularly those born prematurely, are at increased risk of hypothermia (temperature <36°C) owing to their high surface area to volume ratio and the increased evaporative fluid losses from the skin, which is associated with increased morbidity and mortality [26,27]. Drying the baby with prewarmed towels and radiant warmers are not sufficient to prevent heat loss in premature infants and the current guidelines recommend placing newborn infants <28 weeks in a polyethylene wrap or bag immediately after birth keeping the temperature of the delivery room at 26°C [24,28]. Early skin-to-skin contact is a another technique to prevent heat loss in premature newborns [29], a viable option in resource-limited settings. Though the evidence does not suggest an increased risk of hypothermia in preterm infants who have undergone delayed cord clamping (30-60 seconds) [20], there is a potential risk of a drop in core temperature if measures are not taken to prevent heat loss in extreme premature infants during delayed cord clamping especially if the delay exceeds 60-120 seconds. The healthcare providers need to be cognizant of this potential adverse event. Rapid milking of the umbilical cord at birth may provide the benefits of placental transfusion while limiting the risk of hypothermia and requires further study [30].

### Meconium stained amniotic fluid

**Figure 3:**
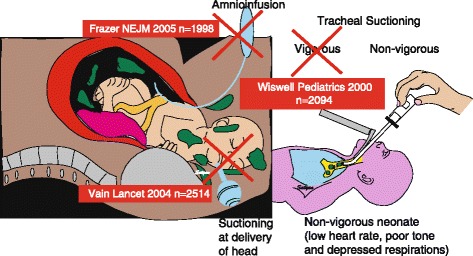
**Management of meconium-stained amniotic fluid (MSAF).** Randomized controlled trials (RCT) with large numbers of mothers and neonates (n – shown in parentheses for each study in a red box) do not support amnioinfusion or oropharyngeal suction following delivery of the head or tracheal suction in the vigorous newborns delivered through meconium-stained amniotic fluid (MSAF). The current guidelines recommend tracheal suctioning only for neonates that are not vigorous and born through MSAF. Copyright Satyan Lakshminrusimha.

The significance and perinatal management of meconium stained amniotic fluid (MSAF) has evolved over time. Routine amnioinfusion, oral/nasal and tracheal suctioning [31-34] were considered to reduce the incidence of meconium aspiration syndrome (MAS) by diluting meconium consistency, decreasing cord compression and removing meconium [35]. Large, multicenter, randomized trials concluded that amnioinfusion, oral, nasal and/or tracheal suctioning in vigorous infants did not alter the incidence of MAS (Figure 3) [7-9].

The Neonatal Resuscitation Program (NRP) protocol for delivery room management no longer recommends tracheal suctioning for vigorous infants [5]. Approximately 20-30% of infants born through MSAF are depressed at birth with an Apgar score of 6 or less at 1 minute of age [36]. Babies exposed to MSAF who have respiratory depression at birth have a higher incidence of MAS [37]. If a baby is born through MSAF, has depressed respirations, decreased muscle tone, and/or a heart rate below 100/min, intubation and direct suctioning of the trachea soon after delivery is indicated before breaths have occurred.

Recent studies in animal models and pilot clinical trials have questioned the benefit of tracheal suctioning in non-vigorous infants born through MSAF. In a lamb model of asphyxia, depression and acute meconium aspiration, tracheal suctioning improved ventilation and oxygenation but did not decrease pulmonary vascular resistance and elevated left atrial pressure possibly as a result of left ventricular dysfunction. The process of tracheal suctioning significantly delayed onset of resuscitation with PPV (47 ± 3 seconds in the no-suction and 146 ± 11 seconds in the suction group, p < 0.001) [Lakshminrusimha et al. Pediatric Research 2014 in press]. A pilot clinical trial evaluating tracheal suctioning in term infants born through MSAF who were not vigorous at birth was presented in an abstract form at the Pediatric Academic Societies meeting in 2014 (E-PAS2014:4680.1). This study randomized 175 neonates to tracheal suctioning (n = 87) and no tracheal suctioning (n = 88). There was no difference in the incidence of death and/or MAS (32% in the tracheal suctioning and 26% in the no tracheal suctioning groups, respectively). These results may influence future guidelines on the approach to non-vigorous infants born to mothers with MSAF.

### Ventilation

#### Fetal lung fluid and first breaths

Active chloride secretion underpins fetal lung liquid secretion and it is critical for lung growth in utero. In late gestation, a rise in fetal glucocorticoids and thyroid hormones readies the sodium absorptive mechanism [38]. Stress of uterine contractions and birth raises fetal epinephrine, which triggers the mechanism responsible for liquid secretion to liquid absorption through the activation of sodium channels in the lung epithelium. After birth, a rise in ambient oxygen augments sodium absorption, which completes the transition to the postnatal state [38]. The process of emptying the lung fluid that begins before birth is augmented by labor and is mostly complete after two hours of independent breathing [39]. During parturition, breathing movements are inhibited, but severe asphyxia can induce gasping movements resulting in the aspiration of meconium during delivery. At birth, breathing results from removal of placental humoral inhibitory factors, cooling, rising carbon dioxide concentrations, and catecholamine surge and induction of genes encoding for substances important for breathing (substance P) [40].

During the first breaths, term infants can generate very high inspiratory (range -28 to -105 cm H_2_O; mean -52 cm H_2_O) and expiratory (range 18-115 cm H_2_O; mean 71 cm H_2_O) pressures to achieve an average inspired volume of 40 mL [41]. First breaths tend to be deeper and longer than subsequent breaths, and are characterized by a short deep inspiration followed by a prolonged expiratory phase through a partially closed larynx, known as expiratory braking [42]. This phenomenon is also observed in infants treated with continuous positive airway pressure (CPAP), frequently slowing and extending the time for expiration [43]. Crying in term and preterm infants immediately after birth also uses expiratory braking and facilitates lung volume recruitment [44]. This observation of spontaneously prolonged initial breaths at birth has prompted investigators to study the effects of a sustained inflation (SI) in apneic infants.

#### Sustained Inflation (SI)

**Figure 4:**
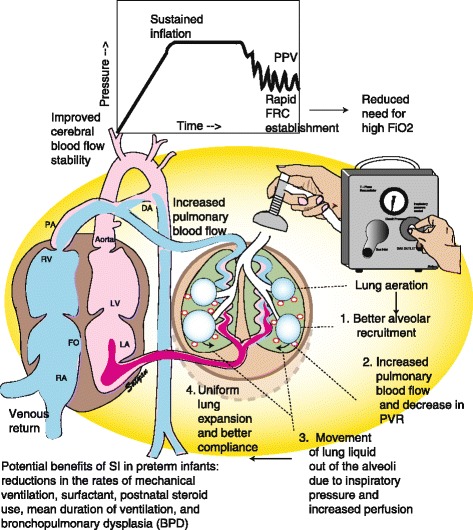
**Sustained inflation (SI) in neonatal resuscitation.** The fluid filled lung of a newborn infant potentially benefits from prolonged inspiratory time and pressure of SI to facilitate (1) alveolar recruitment, (2) increased pulmonary blood flow and decreased pulmonary vascular resistance, (3) movement of lung liquid out of the alveoli, and (4) uniform lung expansion and better compliance. Rapid establishment in FRC results in improved cerebral blood flow stability and reduced need for FiO_2_. DA ductus arteriosus, FiO_2_ fraction of inspired oxygen, FRC functional residual capacity, LA left atrium, LV left ventricle, PA pulmonary artery, PPV positive pressure ventilation, PVR pulmonary vascular resistance, RA right atrium, RV right ventricle. Copyright Satyan Lakshminrusimha.

The optimal ventilation strategy immediately after birth for depressed infants with no spontaneous respiratory effort and liquid-filled lungs is not known. Increasing evidence suggests that SI provided at the onset of resuscitation achieves better lung aeration. Though the duration and pressure of the SI remains to be determined, the new European Resuscitation Council Guidelines recommend SI for the initial ventilation of apneic term and preterm infants [28]. A study in preterm rabbits using SI of 20 seconds, followed by PPV with positive end-expiratory pressure (PEEP), resulted in a rapid increase in FRC and uniformly aerated lungs [45]. In preterm lambs, SI facilitates establishment of pulmonary blood flow immediately after birth and improves cerebral blood flow stability [46]. A study in near term asphyxiated lambs has shown that a single SI of 35 cm H_2_O for 30 seconds immediately after birth improves speed of circulatory recovery and lung compliance [47]. In a premature newborn lamb model, no difference in early markers of lung injury were observed with volume-targeted SI (15 mL/kg volume) when compared to a pressure-limited SI (40 cm H_2_O) [48]. In the clinical setting, Lindner et al. randomized 61 preterm infants <29 weeks gestation to either receive a 15-second SI (20-30 cm H_2_O) or nasal-intermittent mandatory ventilation in the delivery room and reported no difference in adverse effects or the need for mechanical ventilation [49]. Lista et al. have demonstrated reductions in the rates of mechanical ventilation, surfactant, postnatal steroid use, a reduction in the mean duration of ventilation, and bronchopulmonary dysplasia (BPD) in all survivors among the infants that were ≤32 week gestation when an initial 15-second SI followed by nasal CPAP was given in the delivery room [50]. The result of these clinical studies need to be interpreted with caution as SI was only one factor among other changes brought about in the delivery room during these trials. Clinical trials evaluating the efficacy of SI in preterm infants are warranted and are ongoing [51]. The potential benefits of SI are shown in Figure 4.

#### Assisted ventilation and ventilation devices in the delivery room

In many instances, newborns may require ventilatory support shortly following birth, which can be broadly categorized into two groups: (1) respiratory depression and/or inadequate respiratory effort: conditions that lead to hypopnea or apnea including, but not limited to, central nervous system abnormalities (including neuromuscular disorders), asphyxia and/or sepsis, and (2) respiratory distress: conditions that lead to increased work of breathing, including respiratory distress syndrome, retained fetal lung fluid, air-leak syndromes, pleural effusions, and congenital anomalies. The purpose of assisted ventilation is to create and maintain a functional residual capacity (FRC), and to deliver an adequate tidal volume (V_T_) to facilitate gas exchange and stimulate breathing, while minimizing lung injury. This can be achieved by means of CPAP, PPV and SI using any of three resuscitative devices: (1) a T-piece resuscitator, (2) a self-inflating bag or (3) a flow-inflating bag. In spontaneously breathing infants, the increase in intrathoracic pressure during expiration through a narrow glottis can be simulated by applying PEEP by means of CPAP to help them maintain an adequate FRC. Newborns with apnea or severe respiratory compromise may benefit from an initial SI, but are also dependent on a rate and PPV. Unfortunately, knowing the optimal peak inspiratory pressure during PPV to produce an appropriate V_T_ and FRC presents a challenge as V_T_ is dependent on various factors such as (1) spontaneous breathing activity of the infant, (2) lung compliance and resistance, (3) the amount of lung liquid and how quickly it is absorbed, and (4) the inflation-time used [52]. Moreover, during bag-mask ventilation, mask leak can further compromise adequate FRC and V_T_ delivery [53-56]. In the setting of inadequate ventilation, the most recent NRP guidelines devised the MR SOPA acronym (Figure 1) to remind resuscitators to initiate ventilation corrective steps: M (mask adjustment), R (reposition airway), S (suction mouth and nose), O (open mouth), P (pressure increase), A (alternate airway) [5].

Although each ventilation device has its advantages and disadvantages, there is currently no recommendation to guide healthcare providers on the choice of apparatus to use [57,58]. A recent comprehensive, international cluster-randomized crossover study in newborns ≥26 weeks gestation has shown a significant reduction in the number of infants intubated in the T-piece resuscitator group as compared to the self-inflating bag group (17% vs 26%) [59]. A *post hoc* analysis of very low birth weight infants from this study has revealed that infants in the T-piece resuscitator group reaching a HR >100/min by two minutes are less likely to be intubated and less likely to develop BPD [60]. Albeit the disadvantage of self-inflating bags to provide CPAP and PEEP [42], critical in establishing and maintaining an FRC [45], successful ventilation will be dependent on the expertise of providers in using any device available to them. The emphasis should, therefore, be given to adequate training in the use of these devices [5,54].

#### Noninvasive support in the delivery room

Three extensive randomized controlled trials (COIN, SUPPORT and Delivery Room Management Trial) have evaluated the use of CPAP in comparison with immediate intubation and surfactant, showing a trend towards decreased rates of death/BPD and a reduced need for surfactant [61-63]. Two meta-analyses have demonstrated that one additional infant could survive to 36 weeks without BPD for every 25 babies treated with nasal CPAP in the delivery room rather than being intubated [64] and that strategies aimed at avoiding mechanical ventilation have a small but significant beneficial impact on preventing BPD with a NNT of 35 [65]. Following this evidence, in January of 2014, the American Academy of Pediatrics Committee on Fetus and Newborn published a policy statement concluding that “the early use of CPAP with subsequent selective surfactant administration in extremely preterm infants results in lower rates of BPD/death compared with treatment with prophylactic or early surfactant therapy.” [66]. An editorial by Foglia et al. provides an explanation for the modest effects observed with a noninvasive delivery room ventilation strategy [60]. Although the above-mentioned trials were designed to compare CPAP vs routine intubation and surfactant administration, many premature infants require PPV during their initial stabilization after birth. Due to the difficulty in providing adequate mask-ventilation, many infants will require tracheal intubation to achieve effective ventilation.

### Oxygen

Mitochondria provide energy to the living organisms through aerobic metabolism. During this process, mitochondria produce reactive oxygen species as a byproduct that leads to formation of chemical species including highly reactive-free radicals capable of causing functional and structural damage to other cell components. The immature antioxidant defense in newborn and premature infants predisposes this population to higher oxidative stress [67] and cell damage [68]. Beyond the biochemical derangement caused by oxygen toxicity, clinical and animal studies comparing the use of room-air resuscitation to 100% oxygen in asphyxiated subjects have shown an increased time to first cry and a delay in spontaneous respiratory pattern [69,70], implying that pure oxygen may inhibit respiratory activity in newborn infants. Studies in lambs indicate that the decrease in pulmonary vascular resistance at birth can be achieved effectively with optimal ventilation of the lungs with 21% oxygen in control [71], asphyxiated [72], and lambs with pulmonary hypertension and pulmonary arterial remodeling [73]. The understanding of the significance of oxidative stress that emerged during the 1980s and 1990s has contributed to an increased attention to oxygenation levels in newborn babies both in and beyond the delivery room [74]. Since 2010, the new ILCOR guidelines recommend starting term or near-term newborns needing ventilation on air rather than pure oxygen [24]. However, little data exist regarding oxygen supplementation in the period immediately after resuscitation. Though the effects of hypoxemia immediately following birth remain largely unknown, there is considerable evidence pointing to serious deleterious effects of hyperoxemia in the first few hours of life [10,75-77].

#### Asphyxia and hyperoxemia

A recent retrospective analysis of newborn infants with severe perinatal acidemia finds an association between hyperoxemia during the first hour of life and development of hypoxic-ischemic encephalopathy (HIE) [77]. Out of the 120 newborns who had qualified for whole body hypothermia for HIE, 30% had a PaO_2_ > 100 mm Hg during the first hour of their life and were classified as hyperoxemic. The authors proposed that hyperoxemia was associated with a four-fold increased risk of moderate to severe HIE and that the incidence of HIE is directly related to the degree of hyperoxemia. MRI changes compatible with HIE are found in 79% of hyperoxemic newborns and in 33% of normoxic newborns [74]. These results support previous studies that have reported increased risk of poor outcome in babies with hyperoxemia (PaO2 > 200 mmHg) in the first 2 hours of their life [75]. There is also a significant association between fraction of inspired O_2_ (FiO_2_) during the first 6 hours and the adverse effects in cooled babies who had already developed HIE [76]. A recent systematic review and meta-analysis studying the effects of low and high FiO_2_ during the resuscitation of 677 newborn babies ≤32 weeks gestation has shown that reduced mortality approached a significant value when a low FiO_2_ (0.21-0.30) is used, compared to a high FiO_2_ (0.60-1.0) [10]. Healthcare providers need to be aware of the deleterious effects of hyperoxemia, avoid hyperoxygenation and attempt to target normoxia.

The current guidelines recommend supplemental oxygen during CCs. The optimal inspired oxygen during CCs in a neonate with asphyxia and bradycardia or cardiac arrest is not known.

### Chest compressions

The need for CCs is infrequent in neonatal resuscitation, with an estimated occurrence of 0.08% for near-term and term deliveries and a higher frequency (2-10%) in preterm infants [78,79]. Among the survivors who require CCs at birth, many suffer from detrimental neurologic deficits [80,81]. In the event of severe bradycardia or cardiac arrest, CCs are provided to re-establish coronary perfusion pressure (CPP), to revive the heart and achieve return of spontaneous circulation, as well as to provide blood flow to the body. There is still a lack of scientific evidence to support the current recommendations for CCs during neonatal resuscitation. The current guidelines are based on literature published on animal, pediatric and adult research, as well as physiological feasibility and expert opinion [79]. Optimizing the compression to ventilation (CV) ratio, the timing and heart rate nadir to determine when to initiate CCs, and the timing, route of administration and choice of resuscitative drugs still remain to be addressed.

#### Cardiac and thoracic pump theory

**Figure 5:**
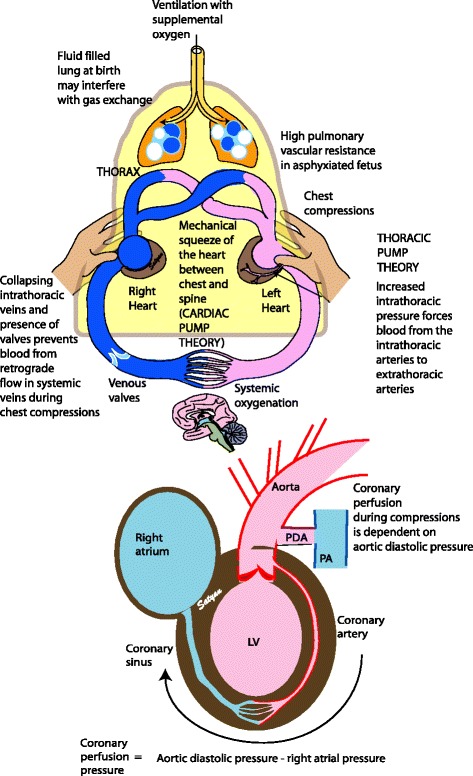
**Chest compressions (CC).** During CCs, the extrinsic pressure on the sternum squeezes the heart against the spine leading to antegrade blood flow (cardiac pump theory). During ventilation, generation of increased thoracic pressure results in arterial blood flow from the thorax because intrathoracic pressure exceeds extrathoracic vascular pressure (thoracic pump theory). Flow is restricted to the arterial-to-venous direction because of collapse of veins at the thoracic inlet and venous valves that prevent retrograde flow. Coronary perfusion pressure (CPP) is a key determinant in return of spontaneous resuscitation and is dependent on aortic diastolic and right atrial pressure. In the presence of a PDA, CCs may be less effective as aortic diastolic pressure may be decreased with blood shunting from the aorta into the pulmonary artery, thus decreasing CPP. LV left ventricle, PA pulmonary artery, PDA patent ductus arteriosus. Copyright Satyan Lakshminrusimha.

The mechanism by which CCs achieve cardiac output can be explained by two theories (Figure 5) [82]: (1) in the thoracic pump theory, external CCs produce an elevation in intrathoracic pressure that is transmitted to the thoracic vasculature. Pressure in intrathoracic arteries is then transmitted to extrathoracic arteries, but jugular venous valves and possible collapse of veins at the thoracic inlet may prevent full transmission of intrathoracic pressure to the extrathoracic veins. This uneven transmission of pressures between the arterial and venous vasculature provides a gradient for antegrade blood flow during CCs; (2) the cardiac pump theory postulates that blood flows through the mechanical squeeze of the heart between the sternum and the spine. The predominant mechanism in the newborn is unclear. In the infant’s more compliant chest, the cardiac pump theory may contribute to blood flow to a greater extent. However, in the setting of sustained inflations and increased intrathoracic pressure, there may be an increased effect through the thoracic pump theory. This effect may partly explain the outcomes in two studies conducted on an asphyxiated piglet model. In the first study, when comparing continuous CCs with asynchronous ventilation to 3:1 CV resuscitation, no difference in return of spontaneous circulation was appreciated [83]. In the second study, when continuous CCs were given during SIs, there was a significant reduction in time to return of spontaneous circulation as compared to the 3:1 CV group [84]. We speculate that by maintaining an increased thoracic pressure with SI, the infant’s chest compliance is decreased and may enhance antegrade blood flow through the thoracic pump mechanism. In addition, with increased pulmonary flow during SI, more blood returns to the heart through the pulmonary veins, effectively increasing ventricle filling volume and thus increasing cardiac output during compressions.

#### Cardiac arrest in the neonate

In contrast to adults, where arrhythmias lead to an abrupt cessation in cardiac output in the setting of well-oxygenated blood, neonatal asystole arises from profound bradycardia as a result of oxygen depletion, carbon dioxide accumulation and increasing lactic acidosis secondary to asphyxia. Oxygen levels remain relatively high in cases of cardiac arrest secondary to arrhythmias. There is a growing body of evidence that under these conditions continuous CCs are associated with improved survival [85-87] and animal models using this approach show improved neurologic outcomes [80-90]. In the asphyxiated neonate with cardiac arrest, due to severe hypoxemia and hypercarbia, ventilation remains critical in establishing return of spontaneous circulation and exclusive CCs in asphyxiated piglet models has not shown to be effective in achieving return of spontaneous circulation [91,92].

#### Chest compression technique and compression to ventilation ratios

Animal and manikin studies have demonstrated that the two-thumb technique provide higher systolic and mean arterial blood pressures, more consistent depth of compressions, improved positioning and less fatigue compared with the two-finger method [93-96]. Even in the setting for emergent umbilical line placement, the provider can continue the two-thumb technique from the head of the bed to leave access at the umbilicus.

Ventilation of the lungs is a critical component of neonatal resuscitation. However, in the presence of extreme bradycardia or cardiac arrest, pulmonary blood flow cannot be sustained and gas exchange does not occur with ventilation alone. Although a combination of PPV and CCs are required for effective resuscitation, the optimal CV ratio remains to be determined. The current 3:1 CV ratio recommended by NRP is an expert consensus, attempting to match the heart and respiratory rates of the newborn. Animal models and manikin studies have compared different compression to ventilation ratios [97-99]. Although a 15:2 CV ratio in an asphyxiated piglet cardiac arrest model has shown statistically higher diastolic pressures, no improvement in return of spontaneous circulation has been achieved without epinephrine [98]. In contrast, a newborn manikin model has shown more consistent depth of compression with a 3:1 CV ratio as compared to 5:1 or 15:2 ratios [99]. The results of a recent study in asphyxiated term piglets comparing the current recommended 3:1 CV resuscitation to continuous CCs with asynchronous ventilation does not reveal a significant difference in the time to achieve return of spontaneous circulation and hemodynamic parameters. However, using the same animal model, the authors have shown significantly improved return of spontaneous circulation with better hemodynamic recovery when continuous CCs were given during SIs as compared to the conventional 3:1 CV group [84]. A clinical study comparing CC during SI to 3:1 CV is currently in progress (ClinicalTrials.gov Identifier: NCT02083705).

### Drugs in the delivery room

Epinephrine has been used in modern cardiopulmonary resuscitation (CPR) since the 1960s. In spite of extensive research on vasopressor use, there is an ongoing controversy regarding the long-term benefits of vasopressor use with possible worse neurologic outcomes as reported in the literature on adult patients [100,101]. Owing to the infrequent use of medications during neonatal resuscitation (<0.1% of live-born deliveries), there is a lack of rigorous scientific evidence for a positive effect of medication in newborns. The recommendations are based on research conducted on animals and adults. Therefore, the role of medications in newborn resuscitation including appropriate dosing, order and route of administration remains controversial [11].

#### Epinephrine in neonatal resuscitation

Epinephrine is a catecholamine with inotropic (increase cardiac contractility), lusitropic (myocardial relaxation), chronotropic (increase heart rate) and vasoconstrictor properties. The vasoconstrictor properties mediated by α-adrenergic receptors are primarily responsible for its effectiveness in CPR [102]. Administration of epinephrine is believed to induce intense peripheral vasoconstriction resulting in elevated systemic vascular resistance and an increase in coronary perfusion pressure to improve coronary flow [103]. Animal studies indicate that CCs alone are inadequate for increasing cerebral blood flow and that the administration of epinephrine results in a higher probability for return of spontaneous circulation [98,104].

Redding et al. have demonstrated for the first time a significant improvement in using 1 mg intravenous (IV) epinephrine (0.1 mg/kg) in asphyxia-induced cardiac arrest in mongrel dogs to achieve return of spontaneous circulation [105]. Human studies following this report do not account for the weight difference and demonstrate return of spontaneous circulation with 1 mg IV epinephrine (~0.01-0.015 mg/kg), which is then extrapolated to neonatal and pediatric patients with dose ranges of 0.01-0.03 mg/kg [11]. The use of high dose IV epinephrine (0.2 mg/kg) in a pediatric swine model has shown to be associated with severe tachycardia, hypertension and higher mortality in the immediate post-resuscitation period [106]. In neonatal lambs, the use of high dose IV epinephrine (0.1 mg/kg) results in reduced stroke volume and cardiac output [107]. In addition there is no evidence to support the use of high dose intravenous epinephrine in adults [100].

#### Endotracheal epinephrine

In a retrospective review, a dose of 0.01-0.03 mg/kg has been first administered by the endotracheal route in 94% of infants requiring epinephrine. With this dose only 32% achieved return of spontaneous circulation. Following a repeat IV epinephrine at the same dose, 77% of the initial non-responders have achieved return of spontaneous circulation. The high frequency of initial endotracheal epinephrine use makes it critical that the recommended dose for endotracheal epinephrine be as effective as possible. Neonatal piglets (2-4 days of age) with induced ventricular fibrillation were resuscitated with 0.01 mg/kg epinephrine or placebo administered into the endotracheal tube, femoral vein, or right atrium. Right atrial and femoral venous administration resulted in optimal peak levels by 1-2 min after administration. No increase in plasma epinephrine concentration was observed after endotracheal epinephrine administration (0.01 mg/kg) [108]. These results confirm that low dose epinephrine by the ETT is not effective.

Barber et al have compared low dose endotracheal epinephrine (0.03 mg/kg) to high dose endotracheal epinephrine (0.07 mg/kg) and IV epinephrine (0.01 mg/kg) in asphyxiated piglets [109]. High dose endotracheal epinephrine is associated with 89% return of spontaneous circulation within 15 min after asystole and all of these piglets were alive 30 minutes after return of spontaneous circulation. When compared to IV epinephrine, high dose endotracheal epinephrine does not cause increased rebound hypertension or tachycardia. If IV access is not readily available, high dose endotracheal epinephrine (0.03 to 0.1 mg/kg) is a reasonable alternative without detrimental effects.

**Figure 6:**
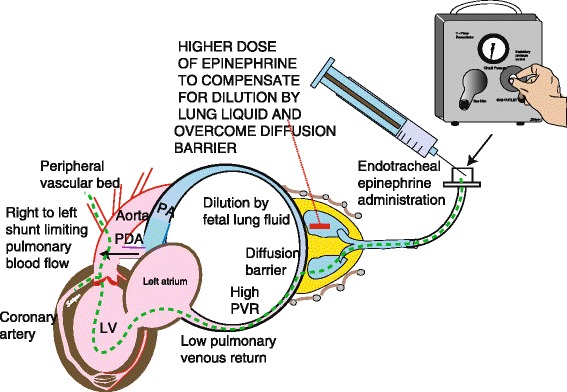
**Endotracheal epinephrine.** Theoretical concerns regarding endotracheal epinephrine administration in perinatal asphyxia. Asphyxia and acidosis lead to a decrease in systemic vascular resistance by dilating the peripheral vascular bed. Coronary perfusion pressure is low. High fetal pulmonary vascular resistance may lead to right to left shunting at the PDA level limiting pulmonary blood flow. Presence of fetal lung liquid may dilute tracheal epinephrine and absorption may be limited by low pulmonary blood flow. The proposed path of intratracheal epinephrine is shown by a hyphenated green line. A higher dose of endotracheal epinephrine may compensate and achieve higher plasma levels. LV left ventricle, PA pulmonary artery, PDA patent ductus arteriosus, PVR pulmonary vascular resistence. Copyright Satyan Lakshminrusimha.

In addition, newborns in the delivery room have unique physiology consisting of fluid-filled alveoli at birth, an open ductus arteriosus and venosus, and the need to transition from fetal to newborn circulation [11,110]. In the presence of asphyxia and acidosis, elevated pulmonary vascular resistance may limit blood flow to the lungs (Figure 6). Studies done in postnatal models such as 1-3 day old piglets that have completed transition may not accurately represent the transitional physiology observed in the delivery room. These physiologic concerns may be partially compensated by using a higher dose of epinephrine via the endotracheal route to compensate for dilution by fetal lung liquid (Figure 6). Lucas et al. have shown that epinephrine levels in pulmonary venous blood increase significantly with hypoxia-associated reduction in pulmonary blood flow [111]. This finding suggests that low pulmonary blood flow may not be the limiting factor for absorption of endotracheal epinephrine. Future studies need to investigate the optimal epinephrine dose to be administered through the endotracheal tube. Early administration of the optimal epinephrine dose by the endotracheal route may promote aortic diastolic pressure, coronary perfusion pressure and hasten return of spontaneous circulation.

Alternate medications such as vasopressin are currently being investigated. Unlike epinephrine, vasopressin is not a direct myocardial stimulant and does not significantly increase myocardial oxygen demand. A recent study in asphyxiated neonatal piglets has shown improved survival, lower post-resuscitation troponin, and less hemodynamic compromise in piglets resuscitated with vasopressin when compared to epinephrine [112]. Though vasopressin has not been shown to be superior to epinephrine in adult CPR [113], its effect in neonatal resuscitation deserves further studies.

### Resuscitation in resource-limited settings

Among the approximately 4 million neonatal deaths annually around the world, up to 70% occur within the first day of life and 99% take place in resource-poor settings [114]. Strategies for prevention of adverse perinatal outcomes in a resource limited setting can be divided into three categories: (1) primary prevention of the insult by adequate fetal monitoring coupled with timely obstetric intervention and referral by traditional birth attendants, (2) secondary prevention after the insult by immediate basic stabilization/resuscitation such as PPV with room air to an infant with respiratory depression, and (3) tertiary prevention of complications in the baby by adequate post-resuscitation care [4]. The ‘Helping Babies Breathe®’ (HBB) program focuses on evidence-based educational programs to teach neonatal resuscitation techniques to resource-limited areas and is being promoted by the AAP, WHO, USAID and the National Institute of Child Health and Human Development (NICHD) [115]. The objective of this program is to train birth attendants in developing countries on the essential skills of newborn resuscitation. In Tanzania, following HBB training in referral and regional hospitals, an increase in babies being stimulated from 44% to 88% resulted in a decrease in bag-mask ventilation [116]. In addition, delayed/physiological cord clamping after the onset of breathing has been shown to reduce the incidence of death/hospital admission in a rural Tanzanian center [117]. Such simple interventions will assist the majority of babies and reduce rates of neonatal mortality.

## Conclusions

Over the past few decades, a tremendous amount of knowledge has been gained in the field of neonatal resuscitation. We have also identified gaps in knowledge on how to best approach and treat newborns in need of aggressive resuscitation. The emphasis should lie on optimizing ventilation, while avoiding lung injury and hyperoxemia. Though sustained inflations may prove to be beneficial, the means by which to best provide positive pressure ventilation to establish and maintain a functional residual capacity, how to best assess ventilation and how to decrease mask leak remains to be determined. In the rare instance that chest compressions and medications are needed to achieve effective return of spontaneous circulation, the optimal compression to ventilation ratio, the timing, route, dose and type of vasopressor still needs to be studied. With the advancement of science and the development of technology, it is expected that healthcare providers will have more reliable tools to reduce the rates of newborn mortality.

In addition to technological advances, the need for further research evaluating strategies for education, dissemination of knowledge and appropriate interventions for resource-limited settings is important. Simple interventions such as delayed/physiological cord clamping, drying and stimulating newborns, and bag-mask ventilation along with programs such as ‘Helping Babies Breathe®’ will have a significant impact on reducing mortality and morbidity secondary to birth asphyxia worldwide.

## Abbreviations

AAP: American Academy of Pediatrics
ACOG: American Congress of Obstetricians and Gynecologists
AHA: American Heart Association
BPD: Bronchopulmonary dysplasia
CCs: Chest compressions
CPAP: Continuous positive airway pressure
CPP: Coronary perfusion pressure
CPR: Cardiopulmonary resuscitation
CV ratio: Compression to ventilation ratio
FRC: Functional residual capacity
HBB: Helping Babies Breathe®’
HIE: Hypoxic-ischemic encephalopathy
ILCOR: International Liaison Committee on Resuscitation
IV: Intravenous
IVC: Inferior venous cava
MAS: Meconium aspiration syndrome
MSAF: Meconium stained amniotic fluid
NNT: Number needed to treat
NRP: Neonatal Resuscitation Program
PEEP: Positive end-expiratory pressure
PPV: Positive pressure ventilation
RCT: Randomized controlled trial
SI: Sustained inflation
USAID: United States Agency for International Development
V_T_: Tidal volume
WHO: World Health Organization
